# Postoperative Hemothorax in an Adult With RIT1-Associated Noonan Syndrome and Reduced Factor XIII Antigen Despite Preserved Activity

**DOI:** 10.7759/cureus.116712

**Published:** 2026-09-22

**Authors:** Bastien Sylvester Claeys

**Affiliations:** 1 Internal Medicine, Solothurner Spitäler, Solothurn, CHE

**Keywords:** diaphragmatic rupture, factor xiii, hemothorax, noonan syndrome, postoperative bleeding, rasopathy, rit1 mutation

## Abstract

Noonan syndrome is a genetically heterogeneous RASopathy associated with congenital cardiovascular abnormalities and a broad spectrum of hemostatic abnormalities. We report the case of an adult man with congenital pulmonary valve disease, previous radiofrequency ablation for Wolff-Parkinson-White syndrome, hepatosplenomegaly, and a clinical suspicion of Noonan syndrome who underwent repair of a previously unrecognized large left-sided diaphragmatic rupture. The defect contained the left colonic flexure, left kidney and adrenal gland, and parts of the stomach and spleen, and was repaired with primary closure and mesh reinforcement. A substantial postoperative left hemothorax developed and required thoracic drainage, followed by persistent and recurrent pleural collections. Subsequent thoracoscopy demonstrated marked inflammatory thickening of the parietal pleura, and pleurectomy was performed for chronic pleuritis. Hematological evaluation demonstrated mildly reduced factor XIII antigen levels on repeat testing, while factor XIII activity remained preserved. Molecular genetic testing subsequently identified a heterozygous pathogenic *RIT1* variant, confirming Noonan syndrome. The available findings do not establish the reduced factor XIII antigen as the cause of the postoperative hemothorax. This case highlights the heterogeneous hemostatic phenotype of Noonan syndrome and the importance of comprehensive perioperative bleeding assessment, including targeted evaluation when clinically significant bleeding occurs.

## Introduction

Noonan syndrome (NS) is an autosomal dominant RASopathy characterized by variable craniofacial, skeletal, lymphatic, and cardiovascular manifestations. *RIT1 *is an established disease-causing gene for NS and is associated with a prominent cardiovascular phenotype [1,2]. Hemostatic abnormalities are also well recognized in NS and may include platelet dysfunction, thrombocytopenia, von Willebrand disease, and deficiencies of individual coagulation factors. A systematic review of 428 patients reported bleeding in 43% of patients and at least one bleeding-related laboratory abnormality in 90% of those with available laboratory data, although a specific bleeding disorder was confirmed in only 45% [3].

Factor XIII is particularly relevant in this context because it stabilizes fibrin, and clinically relevant abnormalities may occur without prolongation of routine coagulation screening tests. A previous case report described factor XIII deficiency in a patient with NS who presented with spontaneous bilateral thigh bleeding and had factor XIII activity of 31% [4]. However, factor XIII antigen concentration and functional activity represent different aspects of factor XIII status, and a reduced antigen level does not necessarily indicate clinically significant functional deficiency when activity is preserved.

The specific relationship between* RIT1*-associated NS, factor XIII abnormalities, and clinically significant postoperative bleeding remains insufficiently characterized. In particular, it is unclear whether an isolated reduction in factor XIII antigen with preserved functional activity has clinical relevance in patients with NS. We therefore report an adult with molecularly confirmed *RIT1*-associated NS who developed a substantial postoperative left hemothorax after repair of a large diaphragmatic rupture. Subsequent hematological evaluation demonstrated repeatedly reduced factor XIII antigen with preserved factor XIII activity. The purpose of this report is to describe the unusual postoperative bleeding event and the associated hematological findings while distinguishing these observations from a causal relationship that cannot be established from a single case.

## Case presentation

A 31-year-old man was evaluated for a previously unrecognized left-sided diaphragmatic rupture. His medical history included a congenital mixed pulmonary valve abnormality with a dysplastic pulmonary valve, mild residual pulmonary stenosis and pulmonary regurgitation, and previous radiofrequency catheter ablation of an accessory pathway for Wolff-Parkinson-White syndrome. A clinical suspicion of NS had been documented before surgery, but molecular confirmation had not yet been performed.

Preoperative abdominal assessment showed hepatosplenomegaly without evidence of advanced chronic liver disease or portal hypertension; liver stiffness on FibroScan was 4.1 kPa, and the spleen measured approximately 19 cm. During diagnostic laparoscopy, a large defect of the left diaphragmatic dome measuring approximately 10 cm was identified. The defect contained the left colonic flexure, upper pole of the spleen, gastric fundus, and left kidney with adrenal gland. Because of the dorsal location of the defect and limited exposure related to the enlarged spleen, the procedure was converted to a median upper abdominal laparotomy. The herniated organs were mobilized and reduced. The diaphragmatic defect was closed primarily and reinforced with an approximately 12-cm circular mesh. There was an estimated blood loss of 150 mL and an intact parietal pleura. 

On postoperative day (POD) 1, radiological assessment demonstrated a significant left hemopneumothorax. A 24-Ch thoracic drain was inserted, and approximately 1300 mL of blood was evacuated from the left hemithorax. The postoperative course was complicated by hypovolemia and acute kidney injury, Acute Kidney Injury Network (AKIN) stage 1.

A subsequent CT examination demonstrated a persistent postoperative collection extending supradiaphragmatically, subdiaphragmatically, and along the liver capsule. Follow-up imaging on POD 8 demonstrated a persistent left-sided hematoma/collection. On POD 22, repeat imaging demonstrated progression of the left pleural effusion. Another repeat CT on POD 34 showed an extensive left pleural effusion with mediastinal shift toward the right (Figure 1). A chest radiograph (Figure 2), also on POD 34, demonstrated extensive left-sided pleural opacity associated with the recurrent pleural effusion. On the same day, approximately 1500 mL of amber-colored left pleural exudate was aspirated.

**Figure 1:**
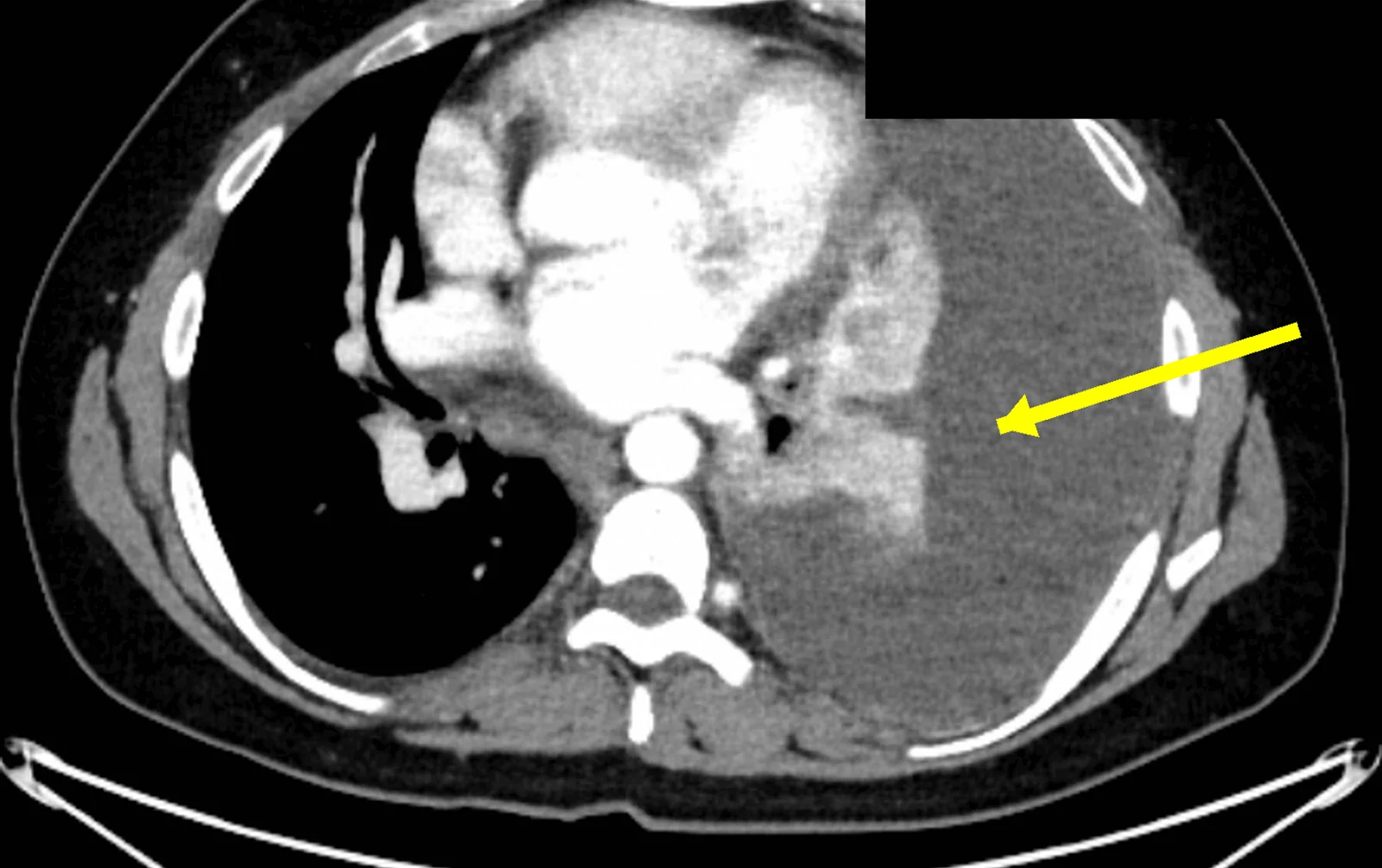
Contrast-enhanced CT of the thorax (axial view) on POD 34 demonstrating a large left pleural effusion with near-complete atelectasis of the left lung and mediastinal shift to the right (arrow) POD: postoperative day

**Figure 2:**
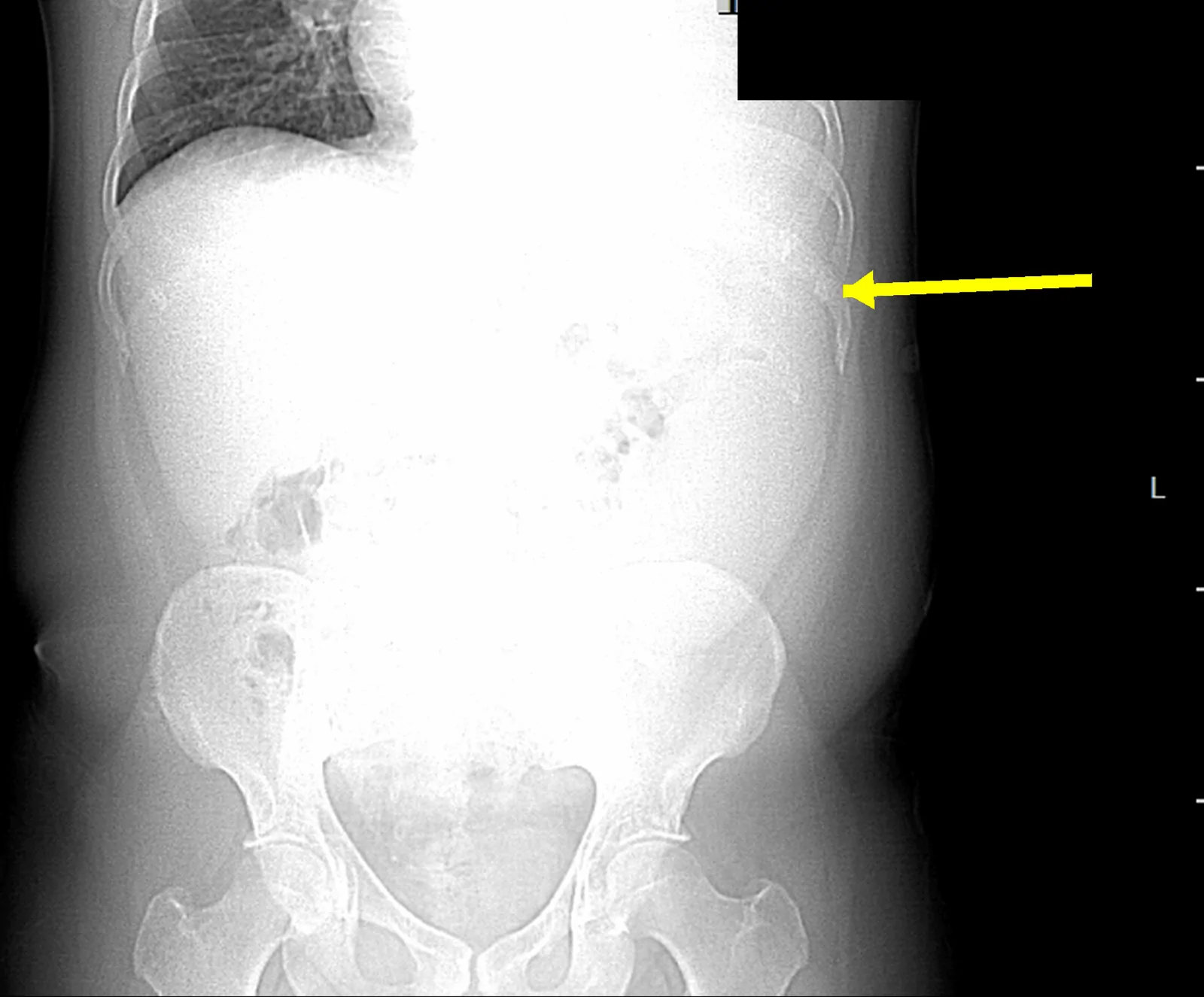
Frontal chest radiograph on POD 34 showing extensive left-sided pleural opacity (arrow) associated with the recurrent pleural effusion POD: postoperative day

Because of recurrent reaccumulation, left thoracoscopy was subsequently performed approximately two months after the initial operation. Thoracoscopy demonstrated marked inflammatory thickening of the parietal pleura without a residual hematoma, while the repaired diaphragm appeared tightly closed. A parietal pleurectomy was therefore performed for chronic pleuritis. Histopathological examination showed chronic granulomatous inflammation and reactive changes without dysplasia or malignancy. Pleural fluid cytology demonstrated reactive mesothelial changes with lymphohistiocytic inflammation and hemosiderin-laden macrophages without malignant cells. 

The postoperative bleeding prompted an extended hematological evaluation. Factor XIII antigen was mildly reduced on repeated testing, with values of 68.5% (reference range, 75.2-154.8%) and subsequently 62.8%. Six months after the initial surgery, factor XIII activity was 75.6% (reference range, 65-150%), whereas factor XIII antigen was 62.8%. A subsequent factor XIII activity measurement, eight months after the initial surgery, was 82%. Thus, the reduced antigen level was accompanied by preserved functional factor XIII activity. The available laboratory assessment therefore did not support a clinically significant functional factor XIII deficiency.

Additional hematological investigations did not demonstrate a clonal lymphoid or myeloid process;* CALR*, *JAK2 *V617F, and *MPL *W515K/W515L mutation testing was negative. Molecular genetic testing using a RASopathy panel subsequently identified a heterozygous pathogenic *RIT1* variant, c.284G>C, p.(Gly95Ala), in exon 5. The variant was classified as pathogenic according to American College of Medical Genetics and Genomics (ACMG) criteria and established the molecular diagnosis of NS [1,2]. During subsequent follow-up, the patient remained clinically stable without documented recurrent major bleeding or impaired wound healing.

## Discussion

This case illustrates the complexity of interpreting clinically significant perioperative bleeding in a patient with NS. The most prominent clinical event was the development of a substantial postoperative left hemothorax shortly after complex diaphragmatic reconstruction. The operation involved extensive adhesiolysis, mobilization and reduction of several herniated abdominal organs, conversion from laparoscopy to open surgery, primary diaphragmatic closure, and mesh reinforcement. Despite the complexity of the procedure, the intraoperative blood loss was only 150 mL, and the parietal pleura was described as intact; approximately 1300 mL of blood was evacuated from the left hemithorax on POD 1.

After the initial postoperative hemothorax, the clinical course was not consistent with persistent active hemorrhage alone. Imaging demonstrated an organized postoperative collection followed by recurrent and progressively enlarging pleural effusion, including mediastinal displacement. Thoracoscopy performed approximately two months after the initial surgery demonstrated marked inflammatory thickening of the parietal pleura without residual hematoma, while the repaired diaphragm appeared tightly closed. Histopathology showed chronic granulomatous inflammation and reactive changes, and pleural fluid cytology showed reactive mesothelial and lymphohistiocytic changes with hemosiderin-laden macrophages but no malignant cells. These findings support a clinical sequence of postoperative hemothorax followed by persistent pleural inflammation and recurrent pleural effusion. The precise anatomical source of the initial postoperative bleeding could not be established.

Bleeding abnormalities are a recognized component of NS. A systematic review by Nugent et al. demonstrated a broad spectrum of hemostatic abnormalities and emphasized that individual patients may have more than one abnormality and that laboratory abnormalities do not necessarily correlate with clinical bleeding [3]. Accordingly, the occurrence of a major bleeding event in a patient with NS warrants consideration of both recognized clinical and laboratory contributors rather than attribution to a single abnormality.

Factor XIII is particularly relevant because abnormalities may not be detected by routine coagulation screening. Kharel et al. described the case of a patient with NS and spontaneous bilateral thigh bleeding who had markedly reduced factor XIII activity of 31% [4]. Our patient differed substantially. Factor XIII antigen was reduced, with values of 68.5% and 62.8%, but measured functional factor XIII activity remained within the laboratory reference range. Six months postoperatively, factor XIII activity was 75.6%, and at eight months postoperative, it was 82%. These findings indicate a quantitative reduction in measured factor XIII antigen in the presence of preserved measured functional activity rather than an established functional factor XIII deficiency. The factor XIII antigen and activity results should therefore be interpreted as complementary rather than interchangeable measurements. However, antigen concentration and functional activity were reported separately, with distinct reference intervals. Also, the two measurements in the laboratory may not have used identical assay methodology, and thus, this report does not infer assay equivalence. More importantly, the preserved factor XIII activity argues against interpreting the reduced antigen concentration alone as evidence of clinically significant factor XIII deficiency.

The temporal relationship is also important. The clinically significant hemothorax occurred immediately after surgery, whereas the factor XIII antigen and activity investigations were performed during the subsequent hematological evaluation. Therefore, these measurements cannot establish the patient's factor XIII status at the exact time of surgery or prove that the laboratory finding contributed to the initial bleeding. The reduced antigen level is nevertheless a reproducible laboratory observation in the context of molecularly confirmed NS.

The case also illustrates why alternative explanations should be considered. The initial operation was technically complex and required conversion to open surgery, extensive adhesiolysis, mobilization of the spleen and other displaced abdominal organs, and repair of a large posterior diaphragmatic defect. The parietal pleura was intact, and the estimated intraoperative blood loss was 150 mL, followed by a clinically significant hemothorax on POD 1. The subsequent course was further complicated by hypovolemia and acute kidney injury, followed by persistent pleural inflammation and recurrent pleural effusion. These operative and postoperative factors provide plausible contributors to the clinical course, although the precise source of the initial hemothorax remains undetermined.

The genetic diagnosis is also relevant. *RIT1*-associated NS has a characteristic clinical spectrum, including frequent cardiovascular manifestations and reported lymphatic abnormalities [1,2,5]. Whether *RIT1*-associated NS has a distinctive hemostatic phenotype remains uncertain. The present observation of reduced factor XIII antigen with preserved activity may contribute to the description of the phenotype, but a single case cannot determine whether this finding is enriched in patients with *RIT1*-associated disease. Additional systematically collected data would be required to establish such an association.

Finally, the patient's subsequent course provides additional clinical context. There was no recurrent major bleeding or impaired wound healing during follow-up. This longitudinal phenotype is consistent with the preserved functional factor XIII activity but does not exclude other mechanisms for the postoperative hemothorax. Overall, the case supports an approach in which perioperative bleeding in NS is assessed comprehensively and laboratory abnormalities are interpreted together with operative findings, imaging, clinical evolution, and longitudinal bleeding history rather than being considered in isolation.

## Conclusions

This report describes a substantial postoperative left hemothorax in an adult with molecularly confirmed *RIT1*-associated NS following complex repair of a large diaphragmatic rupture. Extended hematological evaluation demonstrated repeatedly reduced factor XIII antigen but preserved factor XIII activity. The available findings therefore do not establish a functional factor XIII deficiency or a causal relationship between the factor XIII abnormality and the postoperative hemothorax.

The reduced factor XIII antigen represents a reproducible laboratory finding in this patient, but its clinical significance remains uncertain. The case emphasizes the heterogeneous hemostatic phenotype of NS and the importance of integrating perioperative bleeding history, operative circumstances, clinical evolution, and targeted hemostatic investigations when unexpected bleeding occurs. Further systematic investigation will be required to determine whether reduced factor XIII antigen has clinical or genotype-specific relevance in* RIT1*-associated NS.
